# Lipidomic Profiling of Murine Macrophages Treated with Fatty Acids of Varying Chain Length and Saturation Status

**DOI:** 10.3390/metabo8020029

**Published:** 2018-04-23

**Authors:** Kevin Huynh, Gerard Pernes, Natalie A. Mellett, Peter J. Meikle, Andrew J. Murphy, Graeme I. Lancaster

**Affiliations:** 1 Baker Heart and Diabetes Institute, 75 Commercial Road, Melbourne, VIC 3004, Australia; kevin.huynh@baker.edu.au (K.H.); gerard.pernes@baker.edu.au (G.P.); Natalie.mellett@baker.edu.au (N.A.M.); peter.meikle@baker.edu.au (P.J.M.); andrew.murphy@baker.edu.au (A.J.M.); 2 Faculty of Medicine, Nursing and Health Sciences, Monash University, Melbourne 3004, Australia; 3 Department of Immunology, Monash University, Melbourne 3004, Australia

**Keywords:** lipidomics, macrophages, fatty acids, obesity

## Abstract

Macrophages are abundant within adipose tissue depots where they are exposed to fatty acids, leading to lipid accumulation. Herein, we have determined the effects of various fatty acids on the macrophage lipidome. Using targeted mass-spectrometry, we were able to detect 641 individual lipid species in primary murine macrophages treated with a variety of saturated fatty acids and an un-saturated fatty acid, either alone or in combination. The most pronounced effects were observed for the long-chain saturated fatty acid palmitate, which increased the total abundance of numerous classes of lipids. While other medium- and long-chain saturated fatty acids, as well as the long-chain unsaturated fatty acid, had less pronounced effects on the total abundance of specific lipid classes, all fatty acids induced marked alterations in the abundance of numerous lipid species within given lipid classes. Fatty acid treatment markedly altered overall phospholipid saturation status; these effects were most pronounced for phosphatidylcholine and ether-phosphatidylcholine lipid species. Finally, treatment of macrophages with either palmitate or stearate in combination with oleate prevented many of the changes that were observed in macrophages treated with palmitate or stearate alone. Collectively, our results reveal substantial and specific remodelling of the macrophage lipidome following treatment with fatty acids.

## 1. Introduction

In the last decade, the immune system has been identified as a powerful regulator of adipose tissue (AT) biology and whole body metabolic homeostasis. Specifically, in the lean, insulin-sensitive state, AT is enriched in cells of both the innate and adaptive immune system, including eosinophils, group 2 innate lymphoid cells, regulatory T cells, invariant NK T (iNKT) cells, and M2 macrophages [1]. The concerted actions of these cells contribute to the maintenance of whole body metabolic homeostasis, and their loss from lean AT results in weight gain, impaired glucose metabolism, and insulin resistance [2,3,4,5]. Obesity is characterised by a large expansion in AT, and this occurs concomitantly with a marked alteration in the AT immune cell profile. Specifically, numbers of the aforementioned immune cells present in lean AT are reduced and, in parallel, the numbers of many other immune cells, such as B cells [6], CD8^+^ T cells [7], NK cells [8], neutrophils [9] and M1 macrophages [10], are markedly increased. The collective effect of these alterations is to induce a state of low-grade, chronic inflammation within AT, which disrupts whole body metabolism, as in impaired glucose tolerance and insulin resistance, for example.

Macrophages were the first immune cell to be discovered in AT [10,11], and it is now well established that macrophages are abundant within both lean, insulin sensitive AT, and obese, insulin resistant AT. Due to their proximity to neighbouring adipocytes, AT macrophages are exposed to high levels of fatty acids and other lipids, and lipid uptake by AT-resident macrophages is important for their biological actions. Specifically, lipid uptake and metabolism, particularly of long chain saturated fatty acids by macrophages resident within obese AT, activates inflammatory signaling pathways, potentiating AT inflammation and metabolic dysfunction [12]. In contrast, the macrophages recruited to lean AT during physiological perturbations buffer transient local increases in fatty acid levels [13]. Thus, both physiological and pathological conditions increase fatty acid levels within AT, leading to increased lipid accumulation within AT-resident macrophages [13,14].

Depending on their acyl chain length, e.g., short-, medium-, or long-chain, and saturation status, fatty acids have wide ranging effects on macrophages [15]. For example, long-chain saturated fatty acids are pro-inflammatory; medium-chain fatty acids are relatively benign; and long-chain unsaturated fatty acids often antagonise the pro-inflammatory effects of long-chain saturated fatty acids. Furthermore, certain fatty acids serve as substrates in specific lipid metabolic pathways, or are preferentially stored as neutral lipid. Herein, to gain insight into the effects of increased fatty acid supply on the global cellular lipidome, we have undertaken a comprehensive characterisation of the macrophage lipidome following acute exposure to fatty acids of different acyl-chain length and saturation status.

## 2. Results

**Figure 1:**
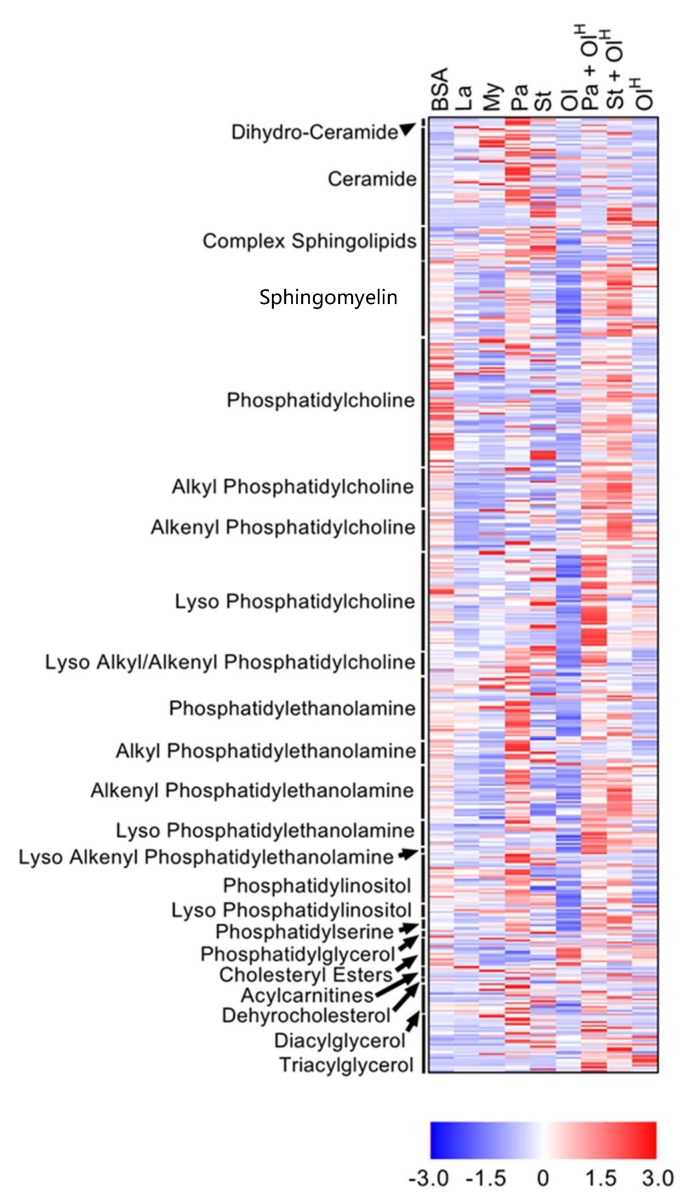
Overview of the effects of fatty acids on the macrophage lipidome. Heat map showing the 504 lipids in which a significant difference existed between at least two groups. Data are presented as Z-score by row, with each individual row representing 1 of the 504 significantly different lipids. Lipid classes are indicated on the *y*-axis: CE, cholesteryl ester; DG, diacylglycerol; LPC, lyso-phosphatidylcholine; LPC-P, lyso-phosphatidylcholine plasmalogen; LPE, lyso-phosphatidylethanolamine; LPE-P, lyso-phosphatidylethanolamine plasmalogen; LPI, lyso-phosphatidylinositol; ether PC, ether phosphatidylcholine; PE, phosphatidylethanolamine; ether PE, ether phosphatidylethanolamine; PG, phosphatidylglycerol; PI, phosphatidylinositol; PS, phosphatidylserine; SM, sphingomyelin; TG, triacylglycerol. Data are from 6 independent mice.

We isolated bone marrow from the hind limb bones of C57BL6/J mice and differentiated these cells into macrophages (bone marrow-derived macrophages (BMDM)), by culturing in L-cell conditioned media for 7 days. BMDM were then treated with 250 μM of the following fatty acids alone, or with a vehicle control (BSA) for 4 h: laurate (12:0; acyl-chain length:number of double bonds), myristate (14:0), palmitate (16:0), stearate (18:0), or oleate (18:1). Because long-chain unsaturated fatty acids are known to antagonise the inflammatory effects of long-chain saturated fatty acids, we additionally co-incubated palmitate or stearate (both at 250 μM) with 1 mM of oleate for 4 h. Finally, we included a group of BMDM that were incubated with 1 mM oleate alone (oleate^High^). Following 4 h of treatment, lipids were extracted from BMDM and extracts analysed using targeted mass spectrometry-based lipidomics. We were able to detect 641 individual lipid species. Differential expression analysis revealed that, of the 641 lipids detected, 504 significantly differed between at least two of the groups. The global effects of the various fatty acid treatments on the BMDM lipidome are shown in Figure 1. A complete list of all 641 lipid species, their mean ± SD in response to the various fatty acid treatments and the associated significance values are included as a Supplementary Microsoft Excel worksheet (Tables S1 and S2).

## 3. Neutral Lipids

**Figure 2:**
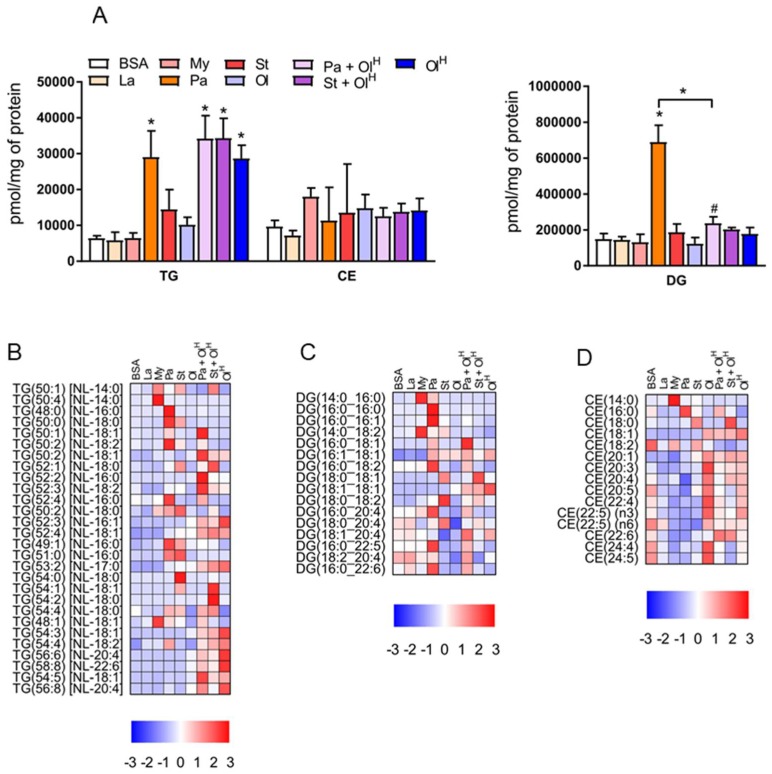
The effects of fatty acids on TG, DG and cholesteryl ester levels. (**A**) Concentrations of total TG (triacylglycerol), DG (diacylglycerol) and CE (cholesteryl esters) in BMDM treated with the indicated fatty acids. Data in A are the mean + SD. Symbols represent statistical significance compared with BSA or, where specifically indicated, between two particular groups at the following α levels: * *p* ≤ 0.001; ^†^ *p* ≤ 0.01; ^#^ *p* ≤ 0.05. (**B**–**D**) Heat maps showing the indicated lipid species within TG (B), DG (C) and CE (D), following treatment with fatty acids. Data are presented as Z-score by row. TG, DG and CE are ordered by sum composition of the acyl chains. Data are from 6 independent mice.

To provide a broad overview of the lipid changes occurring in response to fatty acid treatment, we summed the individual lipid species within given classes to assess total lipid levels. With regards to neutral lipids, which represent the primary stored forms of lipid, significant increases in total triacylglycerol (TG) were observed following treatment with palmitate, palmitate + oleate, stearate + oleate and Oleate^High^ (Figure 2A). In contrast, only palmitate increased total diacylglycerol (DG) levels (Figure 2A). No significant changes in the levels of total cholesteryl esters (CE) were observed between any of the groups (Figure 2A). In contrast to these relatively limited changes in the abundance of total TG, DG and CE, numerous changes in individual lipid species were observed between the groups across all neutral lipid classes (Figure 2B–D). As expected, treatment of BMDM with myristate, palmitate, stearate, or oleate led to an increase in the abundance of neutral lipids containing fatty acyl chains of the corresponding chain length and saturation status (Figure 2B–D). For example, myristate treatment increased levels of the 14:0-containing TG 50:4, DG 14:0_16:0 and CE 14:0; palmitate increased the abundance of numerous 16:0-containing TG and DG species, as well as CE 16:0; and oleate, particularly oleate^High^, increased numerous 18:1-containing TG species (Figure 2B–D). Notably, many of the palmitate- and stearate-induced increases in TG and DG were prevented by co-treatment with oleate (Figure 2B,C).

## 4. Sphingolipids

**Figure 3:**
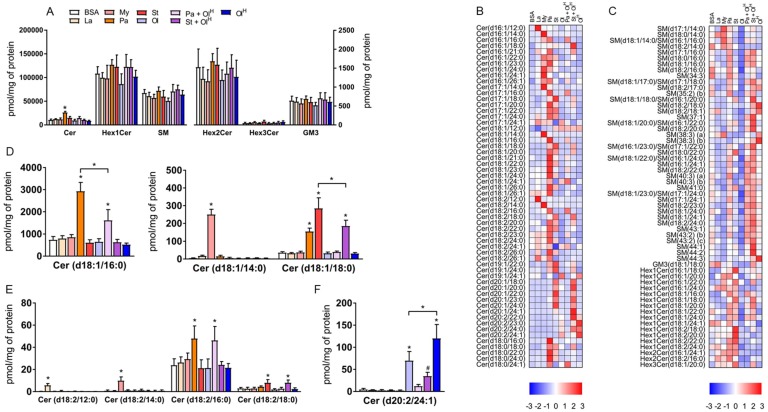
The effects of fatty acids on sphingolipids. (**A**) Concentrations of total ceramide (Cer), ceramide with 1, (Hex1Cer), 2 (Hex2Cer), or 3 (Hex3Cer) sugar moieties, GM3 Ganglioside (GM3) and sphingomyelin (SM) in BMDM treated with the indicated fatty acids. (**B**,**C**) Heat maps showing the indicated lipid species within Cer (**B**) and Hex1Cer, Hex2Cer, Hex3Cer and sphingomyelin (**C**) following treatment with fatty acids. Data are presented as Z-score by row. (**D**–**F**) Bar charts of the concentrations of specific lipid species. Data in A and D–F are the mean + SD. Symbols represent statistical significance compared with BSA or, where specifically indicated, between two particular groups at the following α levels: * *p* ≤ 0.001; ^#^ *p* ≤ 0.05. Data are from 6 independent mice.

Incubation of cells with fatty acids is known to increase the levels of specific sphingolipids, in particular ceramides, which occurs via the *de novo* ceramide synthesis pathway [16]. Excess accrual of ceramides in several cells and tissues, including macrophages, has been linked to inflammation and metabolic dysfunction [16]. At the class level, only palmitate increased total ceramide levels (Figure 3A). The effect of palmitate on ceramide levels is expected as palmitate is the key substrate in the rate-limiting first step in *de novo* ceramide synthesis, the condensation of serine with palmitoyl-CoA. All other fatty acids were without effect on total ceramide levels (Figure 3A). We observed no changes in the total abundance of more complex ceramides, such as glucosylceramides (Figure 3A) containing either 1 (Hex1Cer), 2 (Hex2Cer), or 3 (Hex3Cer) sugar moieties, or GM3 gangliosides (Figure 3A). Similarly, no change in the total abundance of sphingomyelin was observed between groups (Figure 3A). However, these relatively modest alterations in total sphingolipid levels belie a more complex pattern of changes occurring at the level of individual sphingolipid species (Figure 3B,C). Firstly, when BMDM were treated with either laurate, myristate, palmitate, or stearate, the abundance of specific ceramide and Hex1Cer species containing the corresponding acyl group was increased (Figure 3B–E). For example, treatment with the fatty acid laurate (12:0) increased the abundance of Cer(d16:1/12:0) and Cer(d18:2/12:0); treatment with myristate (14:0) increased the abundance of Cer(d17:1/14:0), Cer(d18:1/14:0), Cer (d16:1/14:0) and Cer(d18:2/14:0); treatment with palmitate (16:0) increased the abundance of (Cer18:1/16:0) and Cer(d18:2/16:0); treatment with stearate increased the abundance of Cer(d18:1/18:0), Cer(d18:2/18:0), Cer(d17:1/18:0) and Hex1Cer(d18:1/18:0) (Figure 3B–E). These effects are consistent with the ability of members of the ceramide synthase family of enzymes (CerS1-6) to catalyse the addition of fatty-acyl chains of varying length to the sphingosine base of the ceramide molecule. Secondly, palmitic acid (16:0) is the preferred substrate for serine palmitoyl transferase (SPT), which, following a series of sequential reactions, is metabolised to dihydrosphingosine and acylated by CerS leading to the production of ceramide. Accordingly, treatment of BMDM with the fatty acid palmitate led to a pronounced increase in the abundance of ceramide species containing an 18:1 sphingosine base (2 extra carbons are derived from serine and a double bond is introduced in the final step of ceramide synthesis by dihydroceramide desaturase) (Figure 3B). However, additional fatty acids have been reported to serve as substrates for SPT, notably myristate and stearate [17,18]. Consistently, ceramide species containing either a 16:1 or 20:1 sphingosine base, likely derived from myristate and stearate respectively, were increased in BMDM following treatment with the corresponding fatty acids (Figure 3B). Unexpectedly, we observed an increase in several 20:2 sphingosine base-containing ceramide species in BMDM treated with oleate (Figure 3B,F). Finally, co-incubation of oleate with either palmitate or stearate decreased the levels of many of the ceramide species produced in BMDM treated with either palmitate or stearate alone (Figure 3B,D).

## 5. Phospholipids

**Figure 4:**
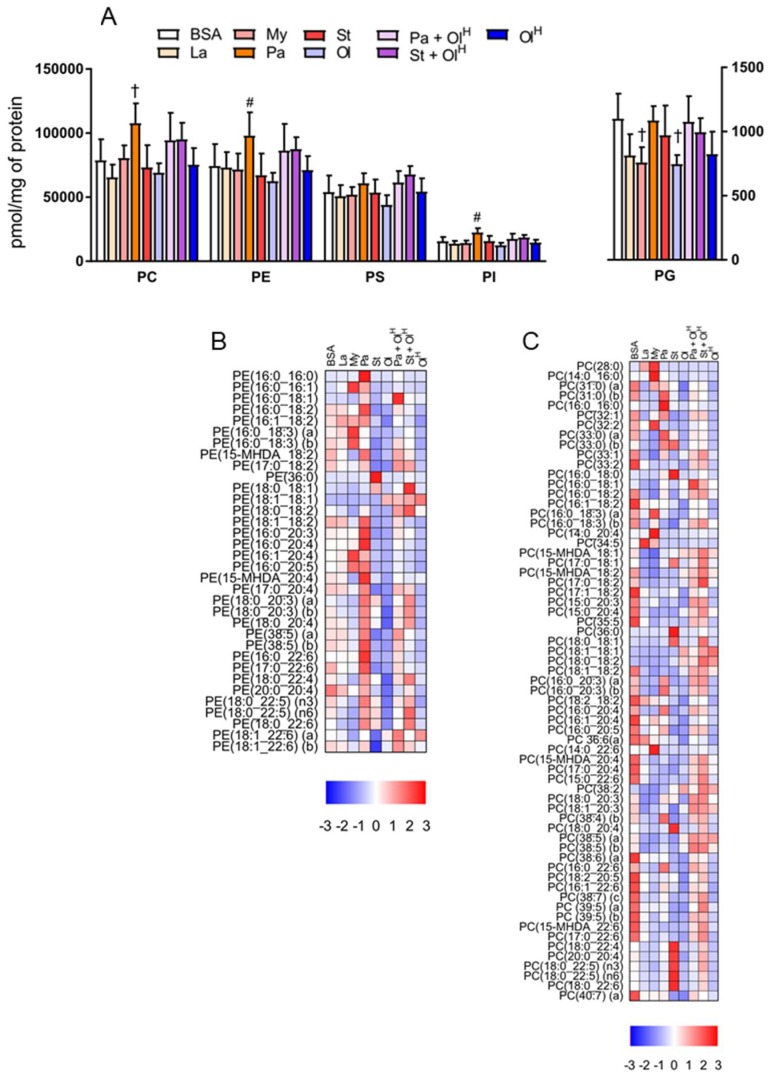
The effects of fatty acids on phospholipids. (**A**) Concentrations of total phosphatidylcholine (PC), phosphatidylethanolamine (PE), phosphatidylserine (PS), phosphatidylinositol (PI) and phosphatidylglycerol (PG) in BMDM treated with the indicated fatty acids. Data in A are the mean + SD. Symbols represent statistical significance compared with BSA at the following α levels: ^†^ *p* ≤ 0.01; # *p* ≤ 0.05. Heat maps showing the indicated lipid species within PE (**B**) and PC (**C**) in BMDM treated with the indicated fatty acids. Data are presented as Z-score by column. Data are from 6 independent mice.

Phospholipids, in particular phosphatidylcholine (PC) and phosphatidylethanolamine (PE), are the principal structural components of cell membranes. We observed relatively modest changes in the total levels of PC, PE, phosphatidylserine (PS), phosphatidylinositol (PI) and phosphatidylglycerol (PG), following treatment with the various fatty acids (Figure 4A). Palmitate treatment led to a significant increase in total PC, PE and PI, while a small decrease was observed in total PG following treatment with either myristate or oleate (Figure 4A). However, while total levels of phospholipids were only modestly changed following fatty acid treatment, the levels of individual phospholipid species within PC and PE were markedly altered (Figure 4B,C). Similar to the situation for ceramides described above, BMDM treated with either laurate, myristate, palmitate, or stearate, accumulated PC and PE species containing the corresponding acyl group (Figure 4B,C). For example, treatment with the fatty acid laurate (12:0) increased the abundance of PC 28:0; treatment with myristate (14:0) increased the abundance of PC 14:0_16:0; treatment with palmitate (16:0) increased the abundance of PE 16:0_20:4 and PC 16:0_16:0; treatment with stearate increased the abundance of PE 36:0 and PC 18:0_20:4); and oleate increased the abundance of PE 18:1_18:1 and PC 16:0_18:1 (Figure 4B,C). Palmitate in particular increased the abundance of numerous PE species (Figure 4B), collectively contributing to an overall increase in total PE. In contrast, stearate and myristate increased the abundance of numerous PC species, while palmitate had quite specific effects on PC, primarily increasing PC 16:0_16:0 (Figure 4C). In many instances the effects of palmitate could be reversed by co-treatment with oleate (Figure 4B,C). While fatty acid treatment led to an increase in the abundance of PC species containing the corresponding acyl-chain, in general, fatty acid treatment decreased the abundance of numerous PC species (Figure 4C). However, although the majority of PC species were decreased following fatty acid treatment, increases in only a select few PC species was sufficient to maintain total PC levels (Figure 4A).

**Figure 5:**
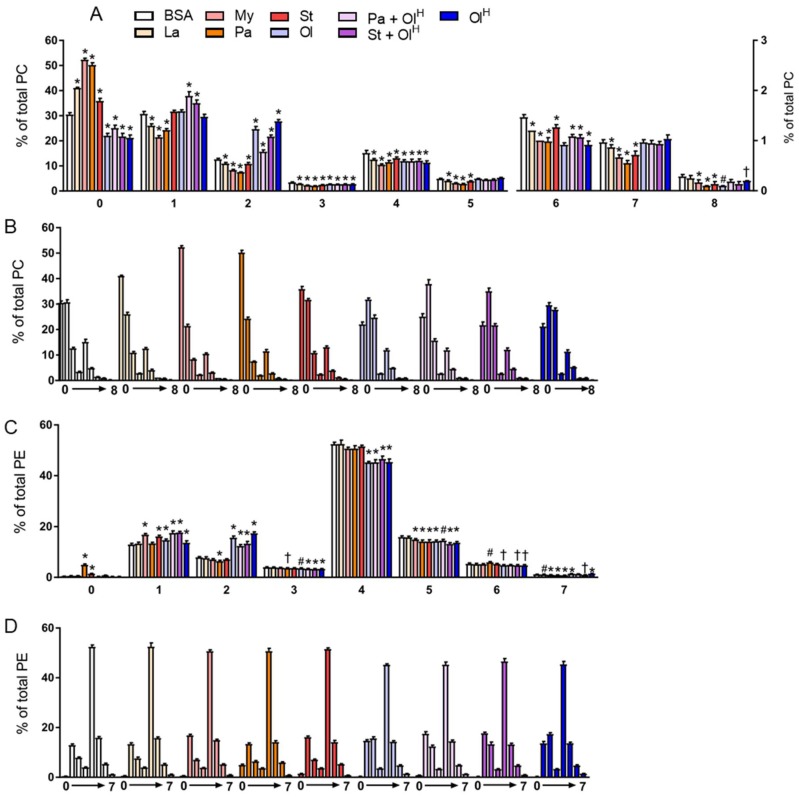
The effects of fatty acids on the saturation of PC and PE. Data in (**A**–**D**) are the mean + SD. Data are expressed in two ways; in A and C the various fatty acid treatments are grouped according to the number of double bonds present, whereas in B and D the data are grouped by the specific fatty acid (or the BSA control). Symbols represent statistical significance compared with BSA at the following α levels: * *p* ≤ 0.001; ^†^ *p* ≤ 0.01; ^#^ *p* ≤ 0.05. Data are from 6 independent mice.

Next, we examined how treatment with the different fatty acids affected the saturation profile of PC and PE. As might be expected, when BMDM were treated with saturated fatty acids, an increase in the abundance of fully saturated PC species was observed (Figure 5A,B). Thus, each of laurate, myristate, palmitate and stearate increased the proportion of fully saturated PC species, from ~30% in untreated BMDM, to 40–50% in BMDM treated with saturated fatty acids. This increase led to a proportional decrease in PC species containing between 1 and 8 double bonds (Figure 5A,B). In contrast, BMDM treated with oleate had a decrease in the proportion of fully saturated PC. Interestingly, only PC species with 2 double bonds were increased upon oleate treatment. The effects of fatty acid treatment on the PE saturation profile was more mixed (Figure 5C,D). Only palmitate and stearate increased the proportion of fully saturated PE, while several fatty acids, although not palmitate, increased the proportion of PE containing 1 double bond. Oleate treatment markedly increased the abundance of PE containing 2 double bonds, and led to a decrease in the proportion of PE containing between 3–6 double bonds (Figure 5C,D). Collectively, these data highlight that exposure to specific fatty acids markedly alters the composition of the cellular phospholipid pool, and that these changes can occur in the absence of pronounced, or indeed any, changes in the total abundance of phospholipids.

## 6. Ether Phospholipids

**Figure 6:**
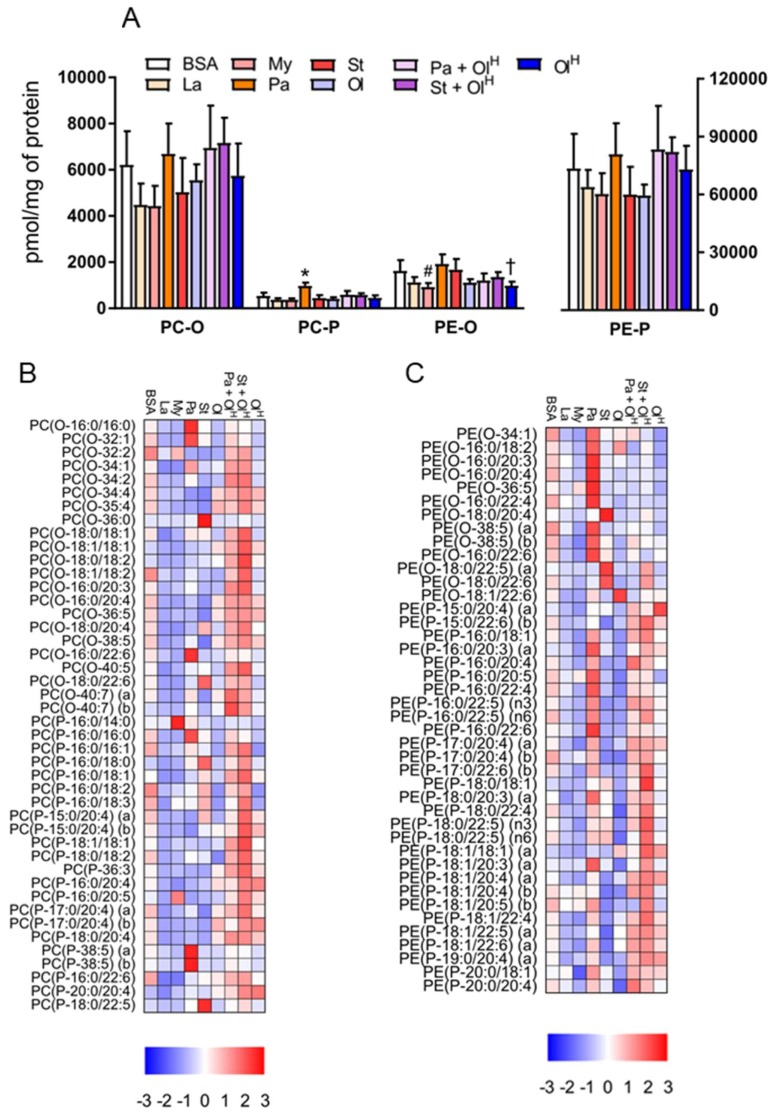
The effects of fatty acids on ether phospholipids. (**A**) Concentrations of total PC-O, PC-P, PE-O and PE-P in BMDM treated with the indicated fatty acids. Data in A are the mean + SD. Symbols represent statistical significance compared with BSA at the following α levels: * *p* ≤ 0.001; † *p* ≤ 0.01; # *p* ≤ 0.05. Heat maps showing the indicated lipid species within PC-O/P (**B**) and PE-O/P (**C**) in BMDM treated with the indicated fatty acids. Data are presented as Z-score by column. Data are from 6 independent mice.

**Figure 7:**
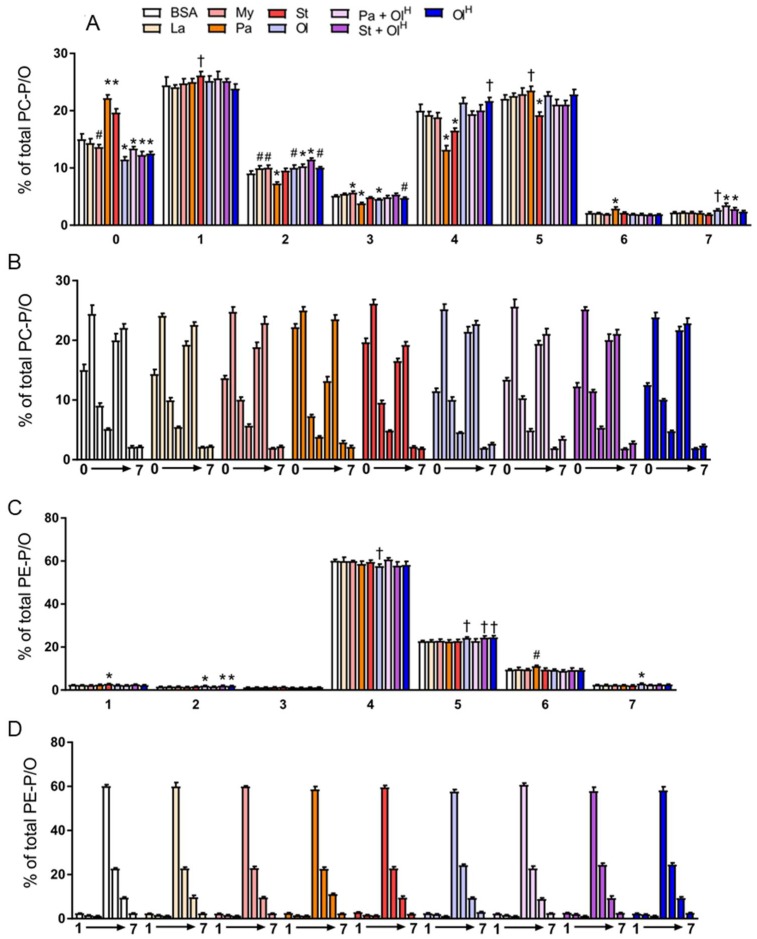
The effects of fatty acids on the saturation of PC and PE ether lipids. Data in (**A**–**D**) are the mean + SD. Data are expressed in two ways: in (**A**) and (**C**), the various fatty acid treatments are grouped according to the number of double bonds present, whereas in (**B**) and (**D**), the data are grouped by the specific fatty acid (or the BSA control). Symbols represent statistical significance compared with BSA at the following α levels: * *p* ≤ 0.001; ^†^ *p* ≤ 0.01; ^#^ *p* ≤ 0.05. Data are from 6 independent mice.

In addition to conventional phospholipids, cells also contain ether phospholipids that are characterised by the presence of an ether-bonded fatty alcohol, as opposed to an ester-bonded acyl chain at the *sn-1* position of the glycerol backbone. Ether lipids can be further classified according to the presence of a *cis*- double bond adjacent to the ether bond. Ether lipids containing this vinyl ether linkage are referred to as plasmalogens, i.e., PC-P/PE-P, while conventional ether lipids are referred to as PC-O/PE-O. The abundance of ether lipids is highly variable between cell types, but they appear to be relatively abundant in myeloid cells, e.g., neutrophils and macrophages. Accordingly, we profiled ether phospholipids within BMDM and characterised how fatty acids influenced their abundance. Fatty acid treatments had no significant effects on the total abundance of PC-O and PE-P, while modest, but significant effects were seen for PC-P and PE-O (Figure 6A). In general, laurate, myristate, stearate and oleate all decreased the levels of numerous ether lipid species, while increasing the levels of a few specific species, typically those containing the acyl-chain with which the cells were being treated (Figure 6B,C). The most marked changes to ether lipid status occurred following palmitate treatment, particularly for PE-P species, many of which were increased by palmitate (Figure 6C). Of note, co-incubation of palmitate with oleate reversed the majority of these changes (Figure 6B,C). A similar effect was also observed for stearate when co-incubated with oleate. Finally, we examined how fatty acid treatment altered the saturation profile of ether phospholipids. Treatment of BMDMs with palmitate or stearate increased the proportion of fully saturated PC ether lipids (Figure 7A,B) and led to a concomitant decrease in the proportion of PC ether lipids containing 2-5 double bonds. Small but significant changes in PC ether lipids were observed following treatment with other fatty acids (Figure 7A,B). PE ether lipids were relatively less affected than PC ether lipids (Figure 7C,D). Treatment with oleate resulted in a small increase in the proportion of PE ether lipids containing 2 and 5 double bonds, while, somewhat surprisingly, palmitate increased the proportion of PE ether lipids containing 6 double bonds (Figure 7C,D). Collectively, while less affected than their conventional PC and PE counterparts, these data demonstrate that ether lipids are altered following exposure to fatty acids.

## 7. Discussion

AT macrophages are exposed to marked fluctuations in the levels of fatty acids. This can occur in pathological conditions, such as obesity, or in physiological settings, such as fasting or cold exposure. The uptake of fatty acids by AT macrophages and their subsequent metabolism in various biosynthetic pathways leads to lipid accumulation [13,14], which can have either deleterious of beneficial effects. Herein, using a number of different fatty acids, including those that are the major products of adipocyte lipolysis (palmitate, stearate and oleate), we have examined how the macrophage lipidome is affected by exposure to specific fatty acids, either alone or in combination. Our data show that fatty acid treatment of murine BMDM markedly alters their lipidome. These changes include alterations to all of the major glycerophospholipids, neutral lipids and sphingolipids, as well as marked changes in phospholipid saturation status. Our results agree with previous work that has demonstrated increases in TG, DG and ceramide in macrophages treated with palmitate [19]. However, using a mass-spectrometry based approach, the findings presented herein provide the most comprehensive analysis of the effects of palmitate on the macrophage lipidome to date. Furthermore, the inclusion of several other fatty acids in our work makes this study one of the most comprehensive examinations of the effects of fatty acids on the cellular lipidome in any cell type.

Of all the fatty acids examined, palmitate had the most pronounced effects on many of the lipids we measured. In particular, palmitate treatment resulted in a marked (~4-fold) increase in total DG and TG abundance. Palmitate also had the most pronounced effects on the sphingolipid ceramide, increasing total ceramide levels due to effects on several abundant ceramide species. As discussed above, this result is not surprising, as palmitate serves as the substrate for *de novo* ceramide biosynthesis. Finally, palmitate was the only fatty acid that led to an increase in the total abundance of PC, PE and PC-P. Palmitate is the canonical pro-inflammatory fatty acid, with numerous investigators using palmitate treatment of cells as a model of lipid oversupply and inflammation. It is likely that palmitate induces inflammation via numerous mechanisms; the accumulation of specific lipids is likely to be critical for its pro-inflammatory effects. Laurate, myristate and oleate are not suggested to be potent inflammatory lipids; indeed, they often antagonise the inflammatory effects of palmitate, and the lipidomic changes induced by laurate, myristate and oleate, are both quantitatively and qualitatively distinct to those induced by palmitate.

Palmitate increases ceramide levels both by serving as a substrate for SPT—and therefore being incorporated into the sphingosine backbone of ceramide—, as well as CerS, which adds a fatty acid to the sphingosine base [16]. The SPT enzyme complex is typically a heterodimer consisting of one subunit each of SPT1 and SPT2. However, an additional SPT subunit, SPT3, as well as a number of small subunits of mammalian SPT (ssSPT), exist and broaden the substrate specificity of the SPT complex beyond palmitoyl-CoA [17,18]. Notably, myristate and stearate, can serve as substrates for SPT and form sphingosine bases with two fewer, in the case of myristate, or two more, in the case of stearate, carbons in the sphingosine backbone; our data provides interesting insights into this issue. Firstly, as expected given the relatively broad specificity of CerS enzymes for fatty acids, we observed an increase in the abundance of ceramide species containing fatty-acyl chains that were presumably derived from treatment with the cognate fatty acid, e.g., an increase in Cer(d18:2/12:0) following laurate treatment. This effect was observed for all the saturated fatty acids used; CerS enzymes do not catalyse the addition of oleate to the sphingosine backbone. However, more interestingly, our results support that myristate and stearate can indeed serve as substrates for the sphingosine backbone, as we observed marked increases in numerous 16:1 sphingosine base-containing (i.e., derived from myristate) and 20:1 sphingosine base-containing (i.e., derived from stearate) ceramide species. Remarkably, and although their abundance was relatively low compared with other ceramide species, we observed an increase in the level of numerous 20:2 base-containing ceramide species. As these same species were not increased in stearate treated macrophages, we presume that the 20:2 sphingosine base of those ceramide species is derived from oleate. Of note, while we did not treat cells with the fatty acid palmitoleate (16:1), quite marked levels of 18:2 sphingosine base-contained ceramide species were observed in BMDM. Given the low level of palmitoleate in the media and the low level of incorporation of oleate to produce 20:2 sphingosine, this would suggest a mechanism other than palmitoleate incorporation is responsible for 18:2 sphingosine synthesis. The structure of plasma sphingadienes has previously been reported to contain double bonds in the C4 and C14 position [20], suggesting an alternate biosynthetic pathway not involving the condensation of serine with palmitoleate, but rather involving a desaturation at the C14 position in addition to the usual C4 position. Where within the biosynthetic pathway of ceramides this desaturation occurs and the enzyme responsible is not currently known.

One of the reasons we selected the fatty acids used in this study was their relative inflammatory effects, with laurate, myristate and oleate being markedly less inflammatory in nature than palmitate and stearate [15,21]. We reasoned that changes in the lipidome induced by both palmitate and stearate, but not by laurate, myristate and oleate, may identify lipids with possible roles in mediating the inflammatory effects of palmitate and stearate. In this regard, palmitate and stearate increased the abundance of numerous over-lapping and distinct ceramide species that were not altered by laurate, myristate, or oleate, supporting previous work that ceramides may be important mediators of long chain saturated fatty acid-induced inflammation [19].

Previous studies have shown that co-incubation of unsaturated fatty acids (e.g., oleate) with long chain saturated fatty acids (e.g., palmitate) prevents the inflammatory actions and of the latter [22,23]. Our findings are consistent with these previous observations, demonstrating that for many of the lipids examined, co-incubation with oleate either attenuated or prevented the effects of palmitate or stearate alone. Interestingly, while the palmitate- and stearate-induced increases in the abundance of numerous ceramide species were prevented by co-incubation with oleate, a significant number of ceramide species, while being increased by both palmitate and stearate treatment, were not altered by co-incubation with oleate; the reason for this differential effect is unclear.

In the present study macrophages, were treated with fatty acids for a relatively short period of time (4 h). We chose this incubation period as we typically find that 2–4 h of treatment is required for long chain saturated fatty acids, such as palmitate, to initiate inflammatory signaling pathways. Accordingly, if the accumulation of specific lipids are important for fatty acid-induced inflammation, which numerous investigators propose to be the case, then their abundance should be increased by 4 h of treatment. However, it is very likely that a more prolonged treatment would magnify many of the effects observed herein, resulting in an even more pronounced alteration of the macrophage lipidome.

To investigate the effects of fatty acids on the macrophage lipidome, we used the commonly adopted method of differentiation in L-cell condition media to generate macrophages. Macrophages generated using this approach are not polarized by specific stimuli, e.g., LPS and cytokines, and can be considered as M0 macrophages, i.e., non-polarized or un-activated. Macrophages exist across a wide range of activation states, which is influenced by their tissue localisation and exposure to specific stimuli [24,25]. Conceptually, at one end of the spectrum are so called M1 macrophages, also referred to as classically-activated macrophages, which are typically present at sites of tissue damage or infection and are highly inflammatory in nature; at other end are M2 macrophages, also referred to as alternatively-activated macrophages, which are located at numerous sites throughout the body and have key roles in tissue homeostasis. Importantly, previous work has found that macrophages found in lean, insulin-sensitive AT are similar to M2 macrophages, while those found within obese, insulin-resistant AT are more akin to M1 macrophages [1]. Importantly, marked differences in cellular metabolism, including lipid metabolism, exist between M1 and M2 macrophages [26]. Specifically, M1 macrophages have an increased reliance on aerobic glycolysis and a decrease in fatty acid oxidation [27], while molecules involved in lipid transport and fatty acid oxidative capacity are increased in M2 macrophages [27]. Thus, we hypothesise that differences in lipid metabolism inherent to AT macrophages resident within either lean, insulin-sensitive or obese, insulin-resistant AT, will likely have a significant impact on the lipidome of the macrophages. In support of this notion, even in the absence of exogenously supplied fatty acids, M0, M1 and M2 macrophages contain different amounts of specific TAG and cholesteryl ester species [27]. Future studies should aim to characterise the lipidomes of macrophages isolated from the AT of lean, insulin-sensitive and obese, insulin-resistant mice. Given that only macrophages present within obese, insulin-resistant AT are of an inflammatory nature, but that macrophages from both lean, insulin-sensitive AT and obese, insulin-resistant AT accumulate lipids, we speculate that the metabolism of fatty acids and their accumulation within specific lipid classes is likely to be different in macrophages from the AT of lean, insulin-sensitive and obese, insulin-resistant mice. As an extension of this idea, it would also be of interest to compare the nature of the lipidomic changes in different cell types following fatty acid exposure. For example, do macrophages accumulate more ceramides than the cells that comprise the tissue in which they reside, e.g., adipocytes and hepatocytes in the case of adipose tissue and liver?

In conclusion, we have demonstrated that acute fatty acid treatment of murine macrophages leads to a marked alteration in the macrophage lipidome. The panel of fatty acids we used herein, which comprised medium and long chain saturated fatty acids as well as long chain un-saturated fatty acids, induce over-lapping and distinct effects on the macrophage lipidome; it is likely that the differences in the formation and accumulation of specific lipids underlie some of the known biological actions of these fatty acids, e.g., inflammatory effects. Hopefully these data serve as a useful resource for other investigators interested in the physiological effects of fatty acids on a variety of cell types.

## 8. Methods

### 8.1. Animals and Cells

C57Bl6/J mice were housed at Alfred Medical Research and Education Precinct (AMREP, Princeton, NJ, USA), Melbourne, Australia. Following CO_2_ asphyxiation of 6 male mice at 12 weeks of age, hind limb bones were collected and bone marrow (BM) harvested by flushing bones with RPMI media. These procedures complied with national guidelines for care and use of laboratory mice, and were approved by an institutional animal ethics committee (AMREP AEC). Following an initial incubation overnight at a density of 1,000,000 cells/mL in RPMI + Glutamax (Life Technologies, 61870-036, Carlsbad, CA, USA), 20% L929-cell conditioned media (vol/vol), 15% fetal bovine serum (vol/vol; FBS) and 1% penicillin/streptomycin (vol/vol) (L-cell conditioned media; LCM), non-adherent BM cells were plated into six-well plates (~1,000,000 cells/well). After 3 days, the volume of media in the well was doubled by adding fresh LCM. Cells were used as BMDM following 7 days of differentiation in LCM. On day 7, the media in the well was aspirated and replaced with media containing RPMI + Glutamax, 5% FBS and 2% BSA (wt/vol; Sigma-Aldrich A6003, Saint Louis, MO, USA). The next day BMDM were treated with fatty acids or vehicle (2% BSA + ethanol). Following treatments, BMDM were washed twice with ice cold PBS and plates stored at −80 °C until further processing.

### 8.2. Fatty Acid Conjugation

Saturated fatty acids were solubilized in 100% absolute ethanol at stock concentrations of 100 mM. Fatty acids were then added to 0.22 μm-filtered RPMI media containing 5% FBS and 2% BSA at a final concentration of 0.25 mM or 1 mM. To facilitate the conjugation of the fatty acids to BSA, these mixtures were gently rocked at 37 °C for ~0.5 h. The following fatty acids were used, all of which were obtained from Sigma-Aldrich: laurate (L4250), myristate (M3128), palmitate (P0500), stearate (S4751) and oleate (O1008).

### 8.3. Lipidomics: Sample Preparation and Analysis

200 μL of ice cold PBS was added to each well and cells scraped from the plate using a rubber scraper. Samples were sonicated with a probe sonicator (Misonix ultrasonix liquid processor with Q Sonica CL5) for 15 s at ~17% amplitude. Total protein concentrations were determined via the BCA method (Thermoscientific, Waltham, MA, USA). Forty micro-liters (20–40 μg of protein) of this lysate was used for subsequent lipid extraction. Lipids were extracted using a single phase chloroform methanol extraction [28] and analysed by LC ESI-MS/MS using an Agilent 1290 liquid chromatography system and Agilent 6490 triple quadrupole mass spectrometer (see Table S3 for conditions). At the time of running the samples in this study, we did not have an acylcarnitine standard. Subsequent to the lipidomic analysis carried out herein, we obtained an acylcarnitine standard (acylcarnitine (16:0) d3) and used this to generate a response factor between acylcarnitine (16:0) d3 and the LPC internal standard (LPC (13:0)). The acylcarnitine values were then divided by the response factor (7.5) to provide acylcarnitine concentrations for this study. The following mass spectrometer conditions were used: gas temperature, 150 °C; gas flow rate 17 L/min; nebulizer 20psi; Sheath gas temperature 200 °C; capillary voltage 3500 V, and sheath gas flow 10 L/min. Liquid chromatography conditions are as follows: ZORBAX eclipse plus C18 column (2.1 × 100 mm 1.8 μm, Agilent) with the thermostat set at 60 °C, with solvent A and B made up of water, acetonitrile and isopropanol (50%/30%/20% and 1%/9%/90% respectively) with 10 mM ammonium formate. Offline characterisation of phospholipid isomers were done in the same chromatographic condition, replacing ammonium formate with 200 mM lithium acetate, using a set of pooled human plasma samples. Negative ionisation mode was used to characterise phosphatidylethanolamine and phosphatidylinositol species, and positive mode with lithium adducts for phosphatidylcholine and sphingomyelin species. Triacylglycerol species are measured as a single fatty acid neutral loss, and their naming is designated as their sum composition with their neutral loss transition, i.e., TG (50:4) (NL-18:2). All data in bar graphs are shown as the mean + the standard deviation. Statistical analysis was performed in SPSS version 22. A 1-way analysis of variance was performed for each of the 641 lipid species we were able to measure, to determine if significant differences existed between the treatment groups. To control the false discovery rate, the Benjamini-Hochberg correction was applied at a false discovery rate of 10%. To determine the location of specific differences between the groups, Tukey’s post hoc test was applied. All figures were created in GraphPad Prism version 7. Experiments were performed in 6 independent mice and data used to create all figures represent an *n* of 6 for each of the treatment groups for all lipid species. No data points were omitted from any of the analyses. The % saturation of specific lipid classes was calculated by firstly determining the total concentration of lipids within a given class that contained a specific number of double bonds, e.g., summing all of the PC lipid species that contain 0 double bonds, and then dividing this value by the total abundance of that same lipid class and multiplying by 100, e.g., dividing the total abundance of PC lipids containing 0 double bonds by the total PC abundance and multiplying by 100.

## Supplementary Materials

The following are available online at http://www.mdpi.com/2218-1989/8/2/29/s1, Table S1: All 641 lipid species and associated P values, Table S2: Post hoc tests, Table S3: Conditions for tandem mass spectrometry analysis of lipid species
