# Supplementary material for: Lipidomic Profiling of Murine Macrophages Treated with Fatty Acids of Varying Chain Length and Saturation Status
Source: Metabolites. 2018 Apr 23;8(2):29. doi: 10.3390/metabo8020029 (PMC6027068; doi:10.3390/metabo8020029)
Supplement: Supplementary file 1 [file metabolites-08-00029-s001.zip › Table S3.pdf]

Supplementary table 3: Conditions for tandem mass spectrometry analysis of lipid species.

Lipid class/subclass	Parent Ion	Fragmentation*	Internal standard	Internal standard (pmol)	Collision Energy (V)
Sphingosine	$[M+H]^+$	NL, 18.0 Da	Sph(d17:1)	50	8
Sphingosine-1-phosphate	$[M+H]^+$	Sphingoid specific^	Sph(d17:1)	50	11-16
Dihydroceramide	$[M+H]^+$	Sphingoid specific^	dhCer(d18:0/8:0)	50	31
Ceramide	$[M+H]^+$	Sphingoid specific^	Cer(d18:1/17:0)	100	23-29
Monohexosylceramide	$[M+H]^+$	Sphingoid specific^	HexCer (d18:1/16:0) d3	50	33
Dihexosylceramide	$[M+H]^+$	Sphingoid specific^	Hex2Cer(d18:1/16:0) d3	50	53
Trihexosylcermide	$[M+H]^+$	PI, *m/z* 264.3	Hex3Cer(d18:1/17:0)	50	57
GM3 ganglioside	$[M+H]^+$	Sphingoid specific^	Hex3Cer(d18:1/17:0)	50	57
GM1 ganglioside	$[M+2H]^{2+}$	PI, *m/z* 366.2	Hex3Cer(d18:1/17:0)	50	9
Sulfatide	$[M+H]^+$	PI, *m/z* 264.3	Sulfatide(d18:1/12:0)	50	56
Ceramide-1-phosphate	$[M+H]^+$	PI, *m/z* 264.3	Cer(d18:1/17:0)	50	29
Sphingomyelin	$[M+H]^+$	PI, *m/z* 184.1	SM(d18:1/12:0)	200	25
Phosphatidylcholine	$[M+H]^+$	PI, *m/z* 184.1	PC(13:0/13:0)	100	21
Alkylphosphatidylcholine	$[M+H]^+$	PI, *m/z* 184.1	PC(13:0/13:0)	100	21
Alkenylphosphatidylcholine	$[M+H]^+$	PI, *m/z* 184.1	PC(13:0/13:0)	100	21
Lysophosphatidylcholine	$[M+H]^+$	PI, *m/z* 184.1 & *m/z* 104.1	LPC(13:0)	100	21
Lysoalkylphosphatidylcholine	$[M+H]^+$	PI, *m/z* 104.1	LPC(13:0)	100	21
Lysoalkenylphosphatidylcholine	$[M+H]^+$	PI, *m/z* 104.1	LPC(13:0)	100	21
Phosphatidylethanolamine	$[M+H]^+$	NL, 141.0 Da	PE(17:0/17:0)	100	17
Alkylphosphatidylethanolamine	$[M+H]^+$	NL, 141.0 Da	PE(17:0/17:0)	100	17
Alkenylphosphatidylethanolamine	$[M+H]^+$	Acyl specific	PE(17:0/17:0)	100	17
Lysophosphatidylethanolamine	$[M+H]^+$	NL, 141.0 Da	PE(17:0/17:0)	100	17
Lysoalkenylphosphatidylethanolamine	$[M+H]^+$	NL, 171.9 Da	PE(17:0/17:0)	100	19
Phosphatidylinositol	$[M+NH_4]^+$	NL, 277.0 Da	PE(17:0/17:0)	100	17
Lysophosphatidylinositol	$[M+NH_4]^+$	NL, 277.0 Da	LPI(13:0)	100	17
Phosphatidylserine	$[M+H]^+$	NL, 185.0 Da	PS(17:0/17:0)	100	25
Phosphatidylglycerol	$[M+NH_4]^+$	NL, 189.0 Da	PG(17:0/17:0)	100	21
Free cholesterol	$[M+NH_4]^+$	PI, *m/z* 369.3	COH d7	10000	23
Cholesteryl ester	$[M+NH_4]^+$	PI, *m/z* 369.3	CE(18:0) d6	1000	10
Other sterol ester derivative	$[M+NH_4]^+$	PI, *m/z* 367.3	CE(18:0) d6	1000	12
Acylcarnitines	$[M+H]^+$	PI, *m/z* 85.1	LPC(13:0)	100	30
Diacylglycerol	$[M+NH_4]^+$	NL, fatty acid	DG(15:0/15:0)	200	21
Triacylglycerol	$[M+NH_4]^+$	NL, fatty acid	TG(17:0/17:0/17:0)	100	21
Alkyldiacylglycerol	$[M+NH_4]^+$	NL, fatty acid	TG(17:0/17:0/17:0)	100	21
Ubiquinone	$[M+NH_4]^+$	PI, *m/z* 197.0	Hex3Cer(d18:1/17:0)	50	17

***** PI, product ion; NL, neutral loss **^** d18:1 base, 264.3. d18:2 base, 262.3, d16:1 base, 236.1, d17:1 base, 250.3, d20:1 base, 292.3, d20:2 base, 290.3.
